# Study of a Fiber Optic Fabry-Perot Strain Sensor for Fuel Assembly Strain Detection

**DOI:** 10.3390/s22239097

**Published:** 2022-11-23

**Authors:** Jianan Jiao, Jianjun Chen, Ning Wang, Jie Zhang, Yong Zhu

**Affiliations:** 1 The Key Laboratory of Optoelectronic Technology & System (Ministry of Education), Chongqing University, Chongqing 400044, China; 2 The Key Laboratory of Electromagnetic Technology and Engineering, Nanchong of Sichuan, China West Normal University, Nanchong 637000, China

**Keywords:** strain sensor, Fabry-Perot, pressurized water reactor

## Abstract

This paper proposes a fiber optic Fabry-Perot (F-P) strain sensing system using non-scan correlation demodulation applied to the health monitoring of the pressurized water reactor’s fuel assembly structures. The structural design and sensing mechanism analysis of the sensor were carried out, and the strain transfer model from the fuel sheet to the strain gauge was established. After the sensor fabrication and installation, the static tests have been conducted, and the results indicate that the sensing system can accurately measure the microstrain with a sensitivity of up to 12.6 nm/με at a high temperature (300 °C). The dynamic testing shows that the sensing system has a good frequency adaptation at 10–500 Hz. Thermal-hydraulic experiments show that the sensing system can run stably in a nuclear reactor, with high temperature, high pressure, and high-velocity flow flushing; additionally, the consistency deviation of the measured data is less than 1.5%.

## 1. Introduction

The internal components of fuel assemblies in pressurized water reactor nuclear power plants are likely to deform under mechanical fatigue due to prolonged scouring by the coolant flow with high velocity, causing safety hazards [1,2,3]. It is an important method to determine the mechanical deformation by measuring the strain in the internal components for the health monitoring of fuel assemblies’ structure. Because of the pressurized water reactors’ high temperature and pressure, the conventional electrical strain sensors cannot stably work for a long time [4,5,6]. Therefore, the fiber optic sensor is considered favorable for its excellent adaptability to high-temperature, high-pressure, and humid environments [7,8,9,10,11].

The current research on fiber optic strain sensors applied in harsh circumstances mainly focuses on fiber grating sensors (FG) and interferometric fiber sensors. In recent years, researchers have performed a considerable amount of research on the simultaneous measurement of strain and temperature, sensitivity enhancement, and the avoidance of cross sensitivity in the field of fiber optic grating sensing. Mihailov et al. decoupled wavelength shifts due to temperature from those due to strain by recording the blackbody radiation level above 650 °C, and proposed a dual strain/temperature sapphire fiber Bragg grating (FBG) sensor [12]. Sridevi et al. coated an etched FBG with reduced graphene oxide, which has enhanced sensitivity for physical parameters such as strain and temperature; this work exhibited a strain sensitivity of 5.5 pm/με (~5 times that of bare FBGs) and temperature sensitivity of 33 pm/°C (~3 times that of bare FBGs) [13]. Gao et al. proposed a sensor based on the few-mode fiber and the FBG; this work utilized the different sensitivities of the spectrum dips to realize the simultaneous measurement [14]. The application of cascade structure or grating coating is the characteristic of these sensors, but they are often complicated, and the stability of FG sensors is challenging to guarantee under the circumstances of pressurized water reactors with high temperature and pressure in this paper. Looking at interferometric fiber optic sensors, miniaturization, special sensitive structures, and large strain measurements have recently become research hotspots. Kaur et al. proposed a microcavity strain sensors, fabricated using a femto-second laser to achieve the sensor thermally stable to sustain operating temperatures as high as 800 °C, which made it possible to apply this sensor in embedded high-temperature environments [15]. In order to improve the sensitivity, the special structure of the sensor strain transfer became the key. Zhang et al. proposed a highly sensitive strain sensor based on helical structure-assisted Mach-Zehnder interference in an all-solid heterogeneous multicore fiber (MCF). This work locally twisted the MCF into a helical structure, allowing the sensor to exhibit good mechanical strength and achieved a sensitivity of up to 61.8 pm/με [16]. To achieve large-range measurements, Xia et.al proposed a single-side sliding F-P cavity with a large measurement range by inserting two photonic crystal fiber on both sides of a quartz capillary with laser welding fixed and freely suspended layouts, respectively, which achieved a range of 9436.66 με at an experimental temperature of 28–1100 °C [17].

However, the currently reported interferometric fiber sensors are difficult to adapt simultaneously to the high-temperature, high-humidity, and high-pressure operating requirements. Therefore, based on the principle of F-P interference, this paper proposes a fiber optic strain sensing system using optical wedge demodulation. The experimental results show that the sensor is capable of measuring the strain of the fuel sheet under the high-temperature and high-pressure environment of the pressurized water reactor with a sensitivity of 12 nm/με, which has the potential to be applied in the structural health monitoring of pressurized water reactor fuel assemblies.

## 2. Method

### 2.1. Sensing System Design

**Figure 1:**
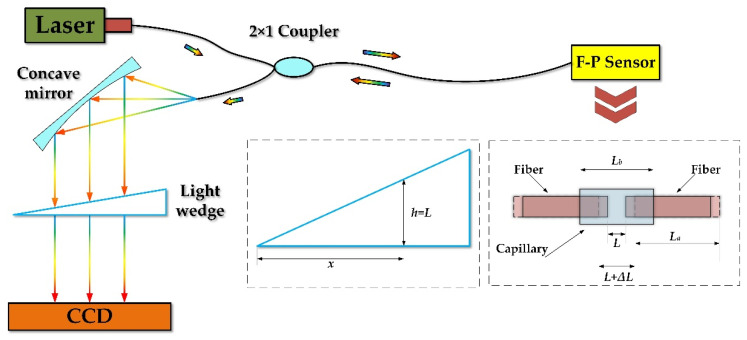
Schematic diagram of the F-P sensing system.

Based on the F-P interference principle, the fiber optic strain sensing system is shown in Figure 1, using an optical wedge for demodulation. The laser light passes through the 2 × 1 coupler into the F-P sensor, where the interference occurs and then returns to the coupler with cavity length information. Afterward, the concave mirror collimates the diverging circular light spot into a parallel line light spot and then enters the light wedge to complete the mutual correlation processing to achieve cavity length matching. The light intensity of the optical signal output from the optical wedge can be expressed as in Equation (1) [18]:

(1) $$I_{out}(\lambda )=A(x)\int_{\lambda_{\min}}^{\lambda_{\max}}\frac{R_{1}+R_{2}+2\sqrt{R_{1}R_{2}}\cos(\frac{4\pi L_{\varepsilon }}{\lambda })}{1+R_{1}R_{2}+2\sqrt{R_{1}R_{2}}\cos(\frac{4\pi L_{\varepsilon }}{\lambda })}\cdot \frac{{\left(1-\sqrt{R_{3}R_{4}}\right)}^{2}}{1+R_{3}R_{4}+2\sqrt{R_{3}R_{4}}\cos(\frac{4\pi x\tan\theta }{\lambda })}\cdot I_{0}(\lambda )d\lambda$$

where *A*(*x*) is the receiving light intensity factor of the detector; *I_out_* and *I_o_* are the output and input light intensities, respectively; the upper and lower integration limits *λ_min_*~*λ_max_* are the spectral bandwidth of the light source; the reflectance at the two ends of the F-P cavity are *R*_1_ and *R*_2_; the reflectance at the two ends of the light wedge are *R*_3_ and *R*_4_; *L_ε_* is the F-P cavity length; *I_o_*(*λ*) is the light intensity of the light source with Gaussian distribution; *x* is the distance from any point in the horizontal direction of the light wedge to the origin.

When the thickness *h* of the light wedge is equal to the F-P cavity length *L_ε_*, the detector can receive the maximum light intensity information. Thus, the F-P cavity’s real-time cavity length can be obtained and the strain of the fuel sheet can be converted.

**Figure 2:**
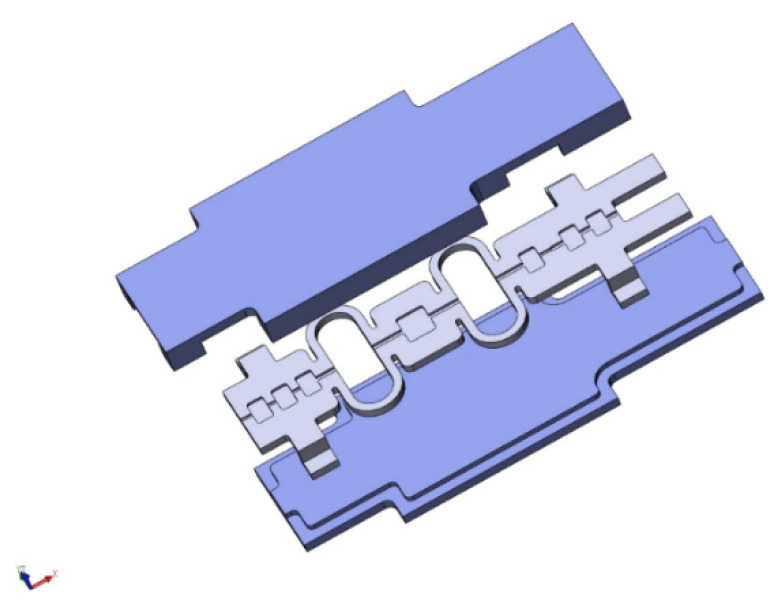
Schematic diagram of the structure of the strain sensor with a double spring ring.

To realize a better fixation of the sensor and a more efficient transfer from fuel sheet strain to the F-P cavity length, a sensor structure was designed, as shown in Figure 2.

The strain sensor comprises two reed rings, three fiber optic clamping platforms and a protective cover on the top and bottom. The strain sensor is equipped with fiber optic clamping grooves on the axes of the reed rings and the fiber optic clamping platform, and the reed rings, delivering strain more efficiently, serve as a strain-sensitive area with the central F-P cavity platform. The sensor and protective cover made of zirconium alloy are combined by welding. When assembling the sensor, the optical fiber is threaded into the F-P cavity, adjusted to an appropriate cavity length, then fastened at the head and tail of the F-P cavity with UV adhesive, and finally, stably fixed and protected on the fiber fixing platform at both ends of the sensor using high-temperature resistant inorganic adhesive.

**Figure 3:**
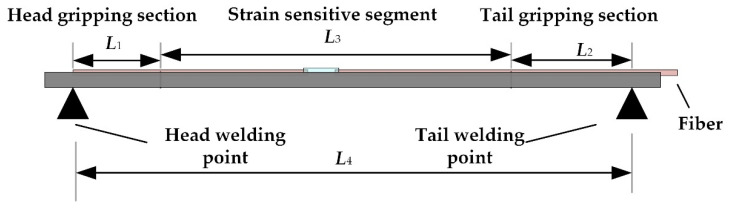
Schematic diagram of the sensor welding clamping model.

The encapsulated strain sensor is clamped to the fuel sheet, and the installation schematic is shown in Figure 3. The F-P sensor is welded to the surface of the fuel sheet, and the principal part of the sensor is suspended after welding to ensure that the fuel sheet strain is transferred to the F-P cavity through the sensor reed ring.

### 2.2. Strain Transfer Analysis

As shown in Figure 3, the head and tail of the base are welded to the fuel sheet, and the length between the welding points is *L*_4_. The head and tail parts clamping section lengths are *L*_1_ and *L*_2_, and the length of the intermediate strain-sensitive section is *L*_3_. When the impact of water flow deforms the outer surface of the fuel sheet, the part between the two welding of the sensor experiences axial deformation accordingly. Assuming the total deformation length of the sensor is Δ*L*_4_, the axial deformation occurring in the head and tail clamping sections on the base are Δ*L*_1_ and Δ*L*_2_, and the axial deformation occurring in the intermediate strain-sensitive section is Δ*L*_3_. The clamping section and the strain-sensitive section are as a whole made with the same material, the elasticity modulus is *E*, and the axial force applied to the clamping end and the strain-sensitive end is equal to *F*, neglecting the effect of the fiber and the adhesive.

(2) $$\Delta L_{4}=\Delta L_{1}+\Delta L_{2}+\Delta L_{3}=\frac{F}{E}\cdot (\frac{L_{1}}{A_{1}}+\frac{L_{2}}{A_{2}}+\frac{L_{3}}{A_{3}})$$

In Equation (2), *A*_1_, *A*_2_*,* and *A*_3_ are the cross-sectional areas of the two clamping and strain-sensitive end, respectively.

The expression for the strain value *ε*_4_ between the two points of welding is:

(3) $$\varepsilon_{4}=\frac{\Delta L_{4}}{L_{4}}=\frac{\Delta L_{1}+\Delta L_{2}+\Delta L_{3}}{L_{4}}=\frac{\varepsilon_{1}L_{1}+\varepsilon_{2}L_{2}+\varepsilon_{3}L_{3}}{L_{4}}=\frac{L_{1}(A_{3}/A_{1})+L_{2}(A_{3}/A_{2})+L_{3}}{L_{4}}\cdot \varepsilon_{3}$$

Using *ε*_3_ in Equation (3) to represent *ε*_1_ and *ε*_2_ and defining *K* as the transfer efficiency of strain from the fuel sheet surface to the strain-sensitive segment, we can obtain Equation (4):

(4) $$K=\frac{\varepsilon_{3}}{\varepsilon_{4}}=\frac{L_{4}}{L_{1}(A_{3}/A_{1})+L_{2}(A_{3}/A_{2})+L_{3}}$$

Equation (4) shows that the transfer efficiency is solely determined by the structure parameters of the sensor.

### 2.3. Thermal Effects

At 300 °C, the optical fibers, capillaries, silicone, and the sensor base are thermally expanded, and the sensor’s initial cavity length will change accordingly. Assuming the length of the optical fibers on both sides is *L_a_*, the length of the capillary tube, silicone rubber, and sensor base are *L_b_*, *L_c_*, and *L_d_*, respectively; *α_a_*, α*_b_*, *α_c_*, and *α_d_* denote the coefficients of linear expansion of the optical fibers, capillary tube, silicone rubber, and sensor base, respectively; when the temperature increases by Δ*T*, the change in the F-P cavity length Δ*L* can be expressed as:

(5) $$\Delta L^{\prime }=2L_{a}^{\prime }+L_{b}^{\prime }+L_{c}^{\prime }+L_{d}^{\prime }=(2\alpha_{a}L_{a}+\alpha_{b}L_{b}+\alpha_{c}L_{c}+\alpha_{d}L_{d})\cdot \Delta T$$

Under the application conditions of high temperature and pressure, as well as water flow scouring, when stress strain occurs on the fuel sheet surface, the F-P cavity length will change to:

(6) $$L^{\prime }{}_{\varepsilon }=L+(2\alpha_{a}L_{a}+\alpha_{b}L_{b}+\alpha_{3}L_{3}+\alpha_{4}L_{3})\cdot \Delta T+\Delta L$$

where *L* is the initial cavity length of the F-P cavity and Δ*L* is the cavity length change due to the measured object’s deformation. Therefore, in the specific application, it is necessary to calibrate the F-P sensor cavity length and normalize the initial cavity length at different temperatures, and then accurately demodulate the variation of the F-P cavity length under a high-temperature, high-pressure, and water flow scouring environment.

## 3. Experiments

### 3.1. Static Testing

**Figure 4:**
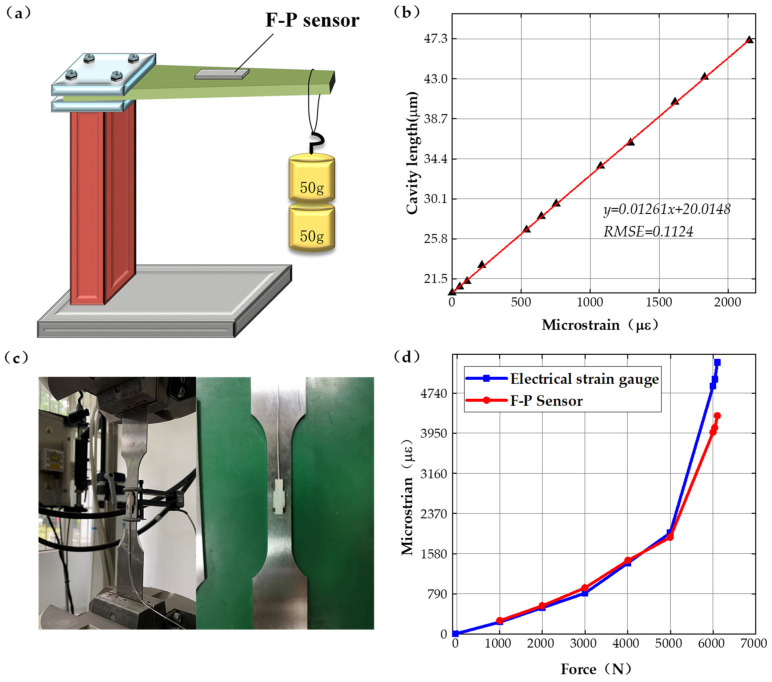
Static experiments. (**a**) Schematic diagram of the calibration experimental system, (**b**) results of the calibration experiments, (**c**) tensile experimental system, and (**d**) results of the tensile experiments.

To verify the static sensor parameters, static testing has been carried out. As shown in Figure 4a, a device for measuring the relationship between the strain and variation of the F-P cavity length was designed in this paper, and the sensor was bonded to the beams of uniform strength using quick-drying adhesive and tested by loading different weights; the results are shown in Figure 4b. When the initial cavity length is 20 μm, and the microstrain (converted from weight mass) varies between 0 and 2200 με, then the cavity length varies in the range of 20–48 μm. The relationship curve between the strain and the F-P cavity length shows a linear variation in the test range, with a sensitivity of 12.6 nm/με.

**Figure 5:**
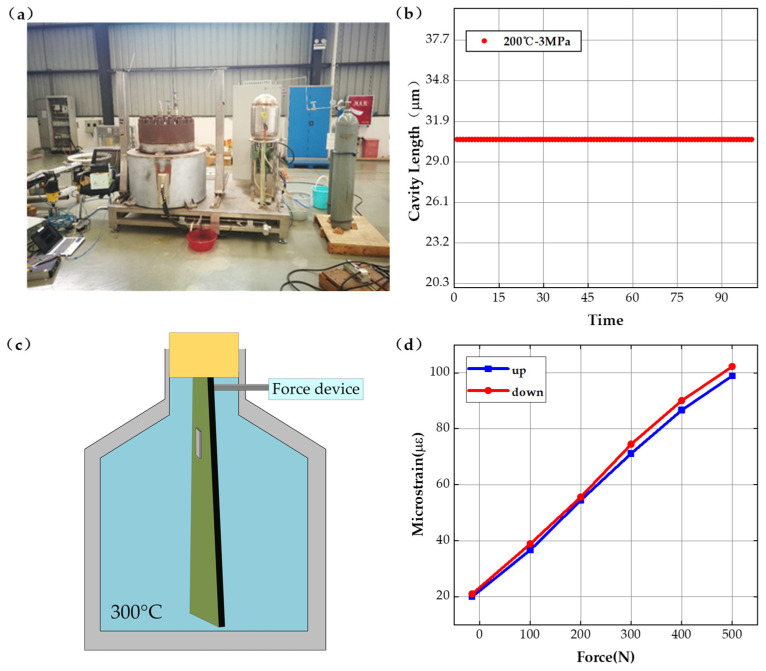
High-temperature experiments. (**a**) Temperature fatigue experimental system, (**b**) temperature fatigue experimental results, (**c**) schematic diagram of high-temperature loading experimental system, and (**d**) high-temperature loading experimental results.

The strain measurement testing installation of the sensing system is shown in Figure 4c, setting the tensile force variation range from 0–7000 N and using the electronic strain measuring gauge and the fiber optic F-P sensor system designed in this paper to perform strain testing, respectively. The relationship curves between tensile force and microstrain are obtained and shown in Figure 4d. When the force varies between 0–5000 N, the maximum deviation between the electronic strain measuring gauge and the fiber optic F-P sensor system is less than 1%. When the force larger than 5000 N, the plastic deformation appears and the tests results have no reference value. The test results indicates that the fiber optic F-P sensing system can measure strain accurately. For testing the initial F-P cavity length variation and its stability at different temperatures and pressures, the initial cavity length has been tested at different temperatures, the experiment setup and tests results are shown in Figure 5a,b. The tests results show that the F-P cavity length offset is less than 0.75% at the determined temperature 200 °C. The temperature does not affect the strain measurement results with accurate calibration of the initial F-P cavity length.

The schematic diagram of the loading and unloading experimental device at high temperature is shown in Figure 5c. When the high-temperature furnace was set at 300 °C, the loading and unloading experimental curves for the equal-strength beams are obtained with the results shown in Figure 5d. The results show that the sensor loading and unloading curve is similar, the maximum measure deviation between the loading and unloading is less than 1.3%, and the sensing system has good stability in the high-temperature environment.

### 3.2. Dynamic Testing

**Figure 6:**
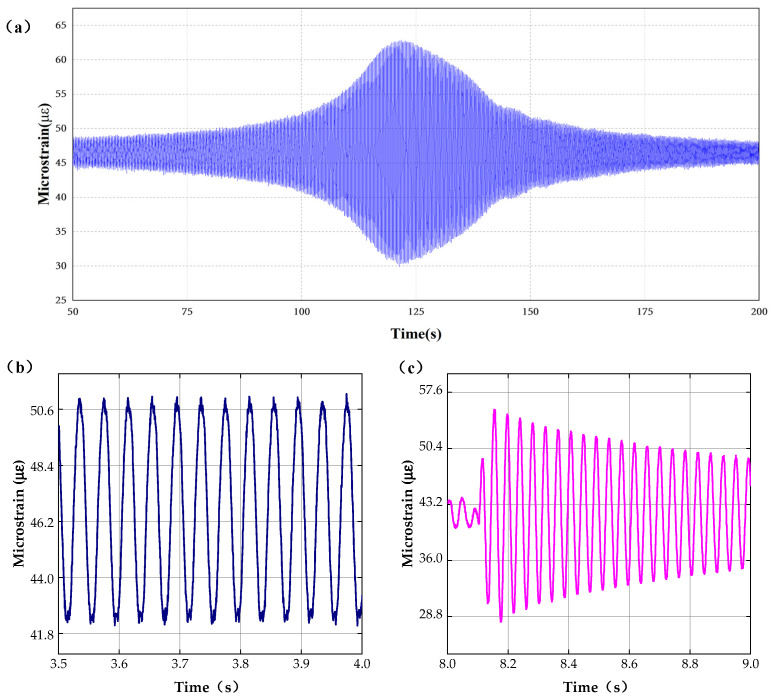
Dynamic experiments. (**a**) Results of frequency sweep experiments, (**b**) results of fixed frequency experiments, and (**c**) results of upside-down vibration experiments.

To verify the dynamic characteristics of the sensor, dynamic response testing at different vibration frequencies has been completed, and the results of the frequency sweep, fixed frequency, and chattering tests are shown in Figure 6.

Figure 6a shows the results of the frequency sweep experiments. In the experiment, the vibration sweep frequency range is 10–500 Hz and the step size of frequency change is 2.5 × 10^−4^ Hz, The results shows that the maximum strain appears at 118.908s which correspond to the 78.1075 Hz. The test results are consistent with the resonant frequency of the fuel sheet.

When the fixed vibration frequency of the control console is set at 25 Hz, the results of the fixed frequency experiments are shown in Figure 6b. The results indicate that the mean value of the output strain is 2.3268 με with a variance of 0.0254, and the output stability of the system strain test is high under the fixed frequency vibration.

Figure 6c shows the results of chattering tests simulating the actual test environment. With a certain degree of elastic deformation of the structure, the test results of free vibration are consistent with the actual free vibration results. The above dynamic tests indicate that the sensing system has ideal dynamic response characteristics and can realize the real-time measurement of fuel sheet strain under dynamic circumstances.

### 3.3. Thermal-Hydraulic Experiments

**Figure 7:**
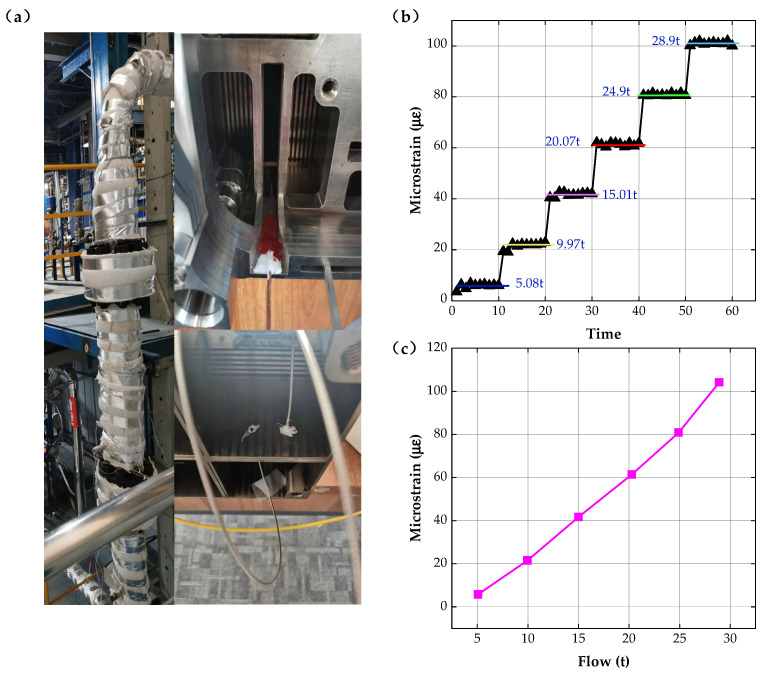
Thermal-hydraulic experiments. (**a**) Experimental environment, (**b**) microstrain variation diagram, and (**c**) strain-flow relationship diagram.

Based on static and dynamic experiments, in the actual thermal-hydraulic environment (high temperature (300 °C), high pressure, and high humidity), the testing of the operating characteristics of the sensing system has been conducted by installing the strain sensors at different positions on the fuel sheet surface and inside the fuel assembly. The experimental environment and analysis results are shown in Figure 7.

In the experiment, the water flow was increased linearly at regular intervals and the microstrain of fuel sheet components under different water flow rates has been tested at 300 °C by sensors at different times; the results are shown in Figure 7b. According to the data, the consistency deviation of the sensing system designed in this paper is less than 1.5% under the condition of high-temperature and high-humidity, which shows the sensor has good stability under the same water flow rate. By removing the initial F-P cavity length change brought by high temperature, the corrected microstrain versus water flow curve is obtained, as presented in Figure 7c; the results accurately reflect the strain change brought by the water flow change.

## 4. Conclusions

This paper proposes a fiber optic F-P strain sensing system based on non-scanning correlation demodulation, and the sensor’s structural design and theoretical analysis are discussed. The static-dynamic characteristics tests and thermal-hydraulic experiments show that the sensitivity reaches 12.6 nm/με, and the use of the elastic spring ring significantly improves the strain measurement sensitivity. The system can constantly work under high temperature (300 °C) and humidity circumstances, and the influence of temperature on the measurement results can be eliminated by calibrating the initial cavity length at different temperatures. Under different water flow rates, the measurement consistency deviation of the system is less than 1.5%, which is promising for structural health monitoring in the harsh environment of pressurized water reactor nuclear power plants.

## Data Availability

Not applicable.
